# Social inequities in vaccination coverage among infants and pre-school children in Europe and Australia – a systematic review

**DOI:** 10.1186/s12889-019-6597-4

**Published:** 2019-03-12

**Authors:** Arzu Arat, Bo Burström, Viveca Östberg, Anders Hjern

**Affiliations:** 1 Department of Medicine, Karolinska Institutet, Stockholm, Sweden; 2 Department of Public Health Sciences, Karolinska Institutet, Stockholm, Sweden; 3 Department of Public Health Sciences, Stockholm University, Stockholm, Sweden; 4 Centre for Health Equity Studies, Karolinska Institutet/Stockholm University, SE-106 91 Stockholm, Sweden

**Keywords:** Vaccination uptake, Immunization, Child primary care services, Equity

## Abstract

**Background:**

Herd immunity levels of vaccine uptake are still not reached in some high-income countries, usually in countries with persisting social inequities in uptake. Previous studies have focused on factors within one health care system. This study takes a broader health care systems approach by reviewing the socioeconomic distribution of vaccination coverage on the national level in light of structural and organizational differences of primary care for children.

**Methods:**

A systematic literature review of socio-economic patterns of uptake of Measles-Mumps-Rubella (MMR) and/or Diphteria-Tetanus-Pertusis (DTP) in population based studies of children 0–5 years of age living in the 30 European Economic Area (EEA) or European Free Trade Association (EFTA) countries and Australia, was carried out using the PRISMA guidelines. The health care system in the countries in the study were categorized by degree of freedom of the primary care provider (hierarchical or non-hierarchical) and whether preventive services were provided in a separate organization (well-baby clinics).

**Results:**

The review identified 15 studies from 10 European countries and Australia that fulfilled the criteria. Although the heterogeneity of the socio-economic indicators did not allow for a conclusive meta-analysis, the study pointed towards lower levels of inequities in primary care models with well-baby clinics. In non-hierarchical primary care organizations that also lacked well-baby clinics, socioeconomic gaps in uptake were often found to be large.

**Conclusion:**

This review indicates that structural and organizational aspects of health care systems for young children are important for equity in vaccine uptake.

**Electronic supplementary material:**

The online version of this article (10.1186/s12889-019-6597-4) contains supplementary material, which is available to authorized users.

## Background

Achieving equity in child health is an important public health challenge on the global level, but also within high-income countries. Equitable access to preventive health services for children has a potential to reduce health inequities during childhood and later in life [1]. A key preventive service in the first years of life is the provision and delivery of vaccinations. Despite significant improvements over the past decades, herd immunity, i.e. the state in which sufficient percent of the population is vaccinated to prevent the spread of disease, is not reached in some high-income countries [2]. Low uptake of vaccine is often associated with persistent social inequities in vaccine uptake [3]. In order to prevent transmission of infectious diseases, herd immunity needs to be reached not only at national level but also within all social strata to prevent the creations of subpopulations with particularly low vaccine coverage where epidemics can start.

Most countries in the European Union, as well as Australia, have publicly funded vaccination programs for children, which aim to overcome financial barriers for families to accessing vaccinations. However, the structure of primary care services and the organization of preventive care may also matter and differ substantially across Europe and Australia. This provides an opportunity for comparative analysis [4].

Based on a classification by Borgueil et al. [5], the organization of primary care services (PCSs) can be grouped into: *hierarchical* and *non-hierarchical*. PCSs with a hierarchical model work under government control and are governed by de-centralized authorities. In countries with this model, governments provide, regulate and fund health services with relatively low freedom for practitioners to set up health care clinics. Spain, Sweden, United Kingdom and the Netherlands are examples of countries that traditionally have had a hierarchical model. PCSs with a non-hierarchical model are characterized by the coexistence of different modes of organization, limited government control and a predominance of solo practitioners with a great degree of freedom where to set up health care clinics. Germany, Austria and France are examples of countries with a non-hierarchical model of primary care services.

Another important distinction across countries is the organization of preventive healthcare services for preschool children. In some contexts (such as in Australia, Netherlands and Sweden), a separate organization is solely responsible for preventive services for children, “well-baby clinics”, where as in others, preventive health services are integrated within the regular primary care services that also provide curative care (such as in United Kingdom). The Well-baby clinics are generally built around a child or public health nurse that work within a team of other child health professionals [6]. These nurses typically schedule visits with families at the clinic according to a preset age dependent schedule, provide telephone counselling and allow for some on-demand visits.

The provision of equitable preventive services depends on multiple structural and organizational factors. Much attention has been paid to the roles of micro-level interventions within one health care system such as the implementation of reminder/recall services, outreach programs, and educational programs for parents and healthcare workers [7]. Factors on the macro level of the national health care system, however, have not been much investigated. Therefore, there is a need to take a broader approach and study socioeconomic distribution of vaccination coverage with a focus on structural and organizational factors of PCSs for children.

The aim of this study was to systematically review the existing empirical studies on socio-economic patterns of vaccination coverage for infants and pre-school children in Europe and Australia and to analyse the findings in the light of structural and organizational differences in primary care at the national level. We hypothesize that having a hierarchical model of primary care organization and presence of well-baby clinics will lead to more equitable services and thus smaller differences in vaccination coverage between socio-economic groups.

## Methods

A systematic literature search was made on July 20, 2017. The following selection criteria, based on Prisma guidelines [8], were used. The search was updated on April 20, 2018 and no new studies were found.

### Search strategies

Pubmed, Embase and Web of Science were used to reach published literature. The final search string consists of three theme blocks, each representing a part of the selection criteria described above: vaccination (outcome), children and infants (population) and socioeconomic determinants (exposure). The theme blocks were then combined by AND command to create a net for capturing articles that contained information on all three theme blocks. The search string used in Pubmed is provided as an example in Additional file 1. This search string was modified according to the specific rules and techniques of each database. An evaluation of the sensitivity of the search string was carried out by testing its capacity to retrieve a list of previously known articles that are highly relevant for the topic of interest.

### Selection criteria

#### Populations and geographic areas of concern

This study is part of a European Horizon 2020 project titled Models of Child Healthcare Appraised (MOCHA) [9]. Countries of the studies to be included in the review were determined by the participating countries in the project. The populations to be included in the review were children aged between 0 and 5, living in one of the European Economic Area (EEA) or European Free Trade Association (EFTA) countries or Australia. The size of the sample population had to be large enough to provide the possibility to detect a 5% difference between population subgroups.

#### Type of studies

Cross-sectional and longitudinal studies with a population-based design were included, thus excluding clinical based studies.

#### Outcome of interest

Studies that reported coverage on Diphteria-Tetanus-Pertusis (DTP) and/or Measles-Mumps-Rubella (MMR) containing vaccines for children aged between 0 and 5 years, and stratified by socio-economic indicators, were eligible for inclusion in the review. In case the outcome was provided in terms of general vaccination status (fully immunized/unimmunized), the study was included if vaccination program under question included MMR and/or DTP.

#### Exposure of interest

Studies that included analyses of vaccine uptake stratified by one of the following four socio-economic indicators were eligible for inclusions: (1) parental income, (2) parental education, (3) parental occupation, and (4) area level socioeconomic status. In case of reporting on both parents separately, the maternal indicators were given priority.

#### Language and time period

Articles in English, German, French, Swedish, Spanish, Danish, Norwegian and Portuguese were included in the study. Studies that had majority of their data collected prior to January 1, 2000 were considered too old to be of interest for the current health care systems and were excluded.

### Screening and selection process

Two researchers screened titles and abstracts of all unique studies. Studies that were selected to be read in full-text, by both researchers, were then reviewed independently. Any disagreement was discussed in detail until a common decision was reached. A hand search was conducted from the reference list of all the included articles as well as the systematic reviews detected through the literature search.

### Data extraction, critical appraisal, and synthesis

Key data from the studies that fulfilled the criteria of the study are presented in Table 1. Heterogeneity between the studies with regards to the socio-economic indicators used made it impossible to perform a meta-analysis. Therefore, studies were synthesized with a narrative approach [10], and grouped based on the organizational and structural factors of primary care services at national level (Table 1). The characteristics of the national health care systems with regards to health care were provided by the country agents of the MOCHA project [11].

**Table 1:** Vaccination coverage in infants and preschoolers with respect to parental SES and primary care models

Author, Year	Setting, Study design, and population	Data source(s)	Outcome(s)	Measurement of parental SES (# of categories)	Overall Vaccination coverage (%)	Key findings (narrative)	Risk of bias
Hierarchical with well-baby clinic							
Pearce, 2015 [19]	Australia, National Sample, Cross-sectional 2004, *n* = 4994	Survey and health records	MMR and DTP combined as part of a series of vaccinations	- Area Level SES (5) - Parental Income (4) - Maternal Education (6)	90.7	There was no association between being unvaccinated and area level SES or parental income. Compared to the highest educated group, the odds of being non-vaccinated was 1.63 (1.04–2.55) for the lowest educated group. There was no significant difference for the rest of the groups.	Medium
van Lier, 2014 [16]	Netherlands, National sample, Cross-sectional 2005–2006, *n* = 180 456	National register	MMR and DTP combined as part of a series of vaccinations	Area level SES (4)	94.5	Areas with lowest SES had only slightly lower vaccination uptake (a difference of 1.9%) compared to areas with highest SES. Higher SES area was associated with higher odds of full vaccination uptake, and lower SES area was associated with lower uptake.	Low
Wallby, 2013 [18]	Sweden, Regional sample (Uppsala), Longitudinal 1998–2006, *n* = 25 024	Regional register	MMR separately and DTP as part of a series of vaccinations	Parental income (4)	92.9 (MMR) 98.5 (DTP)	Both for MMR and DTP only the lowest SES group had slightly lower vaccination rates, 3.4 and 1.9% difference respectively, when compared to the highest SES group. Overall, the findings showed no influence of parental income on vaccination uptake.	Low
Hierarchical without well-baby clinic							
Borras, 2007 [15]	Spain, regional sample (Catalonia), Cross-sectional 2003–2004, *n* = 630	Telephone survey and vaccination cards	MMR and DTP combined as part of a series of vaccinations	-Maternal education(2) -Parental occupation(2)	95.4	Maternal education variable was dichotomized based on university degree and the odds of being vaccinated was 1.84 (1.01–3.33) for higher educated (with a 6%more coverage rate). No association was found for parental occupation.	Medium
Jessop, 2010 [36]	Ireland, Regional sample (Dublin and Galway), Cross sectional 2007, *n* = 749	Survey and health records	MMR	- Maternal education (2) - Parental income (2)	88.7	Children of mothers with higher level of education had 2.1% lower immunization coverage compared to mothers with secondary or lower level education. Their odds of lacking MMR immunization was 1.48 (1.01–2.04) Immunization coverage was 8% lower for children in families that < 300£ per week, compared to families with an income > = 300£. The odds of lacking MMR vaccination was 1.60 (1.35–1.90) for children of parents with low income.	Medium
Doherty, 2014 [21]	Ireland, National sample, Cross sectional 2008–2009, *n* = 9581	Survey	DTP as part of a series of vaccinations	- Maternal Education (2) - Parental occupation (4)	92.0	Neither maternal education nor household occupation were found to be associated with risk of non-vaccination.	Medium
Pearce, 2008 [20]	United Kingdom, National sample, Longitudinal 2000–2002, *n* = 14 578	Survey and health records	MMR	Maternal Education (7)	88.6	Children of mothers with A/AS level or degree had higher risk of being unimmunized when compared to mothers with no education. There was no significant association for other groups.	Medium
Hungerford, 2016 [17]	United Kingdom, Regional sample (Liverpool), Longitudinal 1997–2012, *n* = 62 689	Regional register	MMR	Area level SES (5)	88.4	The least deprived SES group had 6% higher vaccination coverage compared to the most deprived SES group. The risk of being unimmunized for MMR increased linearly with increasing deprivation.	Low
Non-hierarchical with well-baby clinic							
Theeten, 2007 and Vandermeulen, 2008 [12, 13]	Belgium, Regional sample (Flanders), Cross sectional 2005, *n* = 1349	Survey	MMR	- Parental income (5) - Maternal Education (3)	94.0	No significant association was found between family income and MMR vaccination. Parental education was found to be an insignificant factor for MMR coverage.	Medium
Non-hierarchical without well-baby clinic							
Fonteneau, 2013 [22]	France, National sample, Cross sectional *n* = 21 346	Survey and health records	MMR	Parental Occupation (7)	43.3	The group with lowest vaccination coverage was children of farmers (33%). Rest of the groups had 8–13% higher vaccination coverage. However, only three groups (intermediate professions, employees and qualified workers) had significantly higher odds of being vaccinated (OR 1.4), when compared to the children of farmers.	Medium
Mikolajczyk, 2008 [24]	Germany, Regional sample (Bavaria), Cross sectional 2004–05, *n* = 2043	Survey and health records	MMR	Parental Education (3) 1. both parents with low education, 2. One parent with high education 3. Both parents with high education.	93.0	Almost 9% of the children with two high-educated parents and 5% of children with only one highly educated parent were unimmunized. Compared to the group with both parents having high education, the other two groups had lower odds of being unimmunized for MMR. The association was only significant for the children with one parent having high education.	Medium
Poethko-Muller, 2009 [25]	Germany, National Sample, Longitudinal 2003–06, n = 14 826	Survey data and vaccination cards.	MMR (Measles)	Parental education (3)	93.6	Children with high SES background were 3% less vaccinated compared to children in low and mid SES group. Adjusted Odds ratios showed no association between socioeconomic status and vaccination.	Medium
Rosenkotter, 2012 [37]	Germany, Regional sample (North Rhine-Westfalia), Cross sectional 2007, *n* = 52 171	Survey and school-screening program	MMR and DTP combined as part of a series of vaccinations	Parental Education (3)	41.3	Compared to the group with high educated parents, children of parents with low education had 6% lower coverage and double the odds of having incomplete vaccination uptake. There were no significant differences between children with high and medium educated parents.	Medium
Danis, 2010 [23]	Greece, National Sample, Cross sectional 2004–05, *n* = 3878	Survey and vaccination card	MMR and DTP combined as part of a series of vaccinations	Maternal education (4)	63.8	65% of children of mothers with high-school degree and 69% of children of mothers with university degree were completely vaccinated as compared to 54% of children of low educated mothers. Children of mothers with a degree of high school or above had higher odds of being vaccinated compared to the children of mothers with less than 9 years of education.	Medium
Anello, 2017 [26]	Italy, 2 Regional samples (Friuli-Venezia/Giulia [FVG] and Emilia-Romagna [ER]), Longitudinal 1995–2011, *n* = 48 454	Regional register	MMR (separate) and DTP (as part of a series of vaccinations)	Maternal Education (3)	FVG: MMR(89.5) DTP (98.2) ER: MMR (87.9) DTP (97.7)	For MMR, the coverage overall was similar across the groups. In FVG, children of mothers with less than high school education had 3% higher vaccination rates compared to mothers with university degree. In both regions, children of mothers with university degree had higher risk of being unvaccinated when compared to the lowest educated group. For DTP, the coverage was same across all groups. In FVG region, children of mothers with high school degree or higher had risk of being unvaccinated.	Low

A summary table was constructed (Table 2) showing number of results for each association between socio-economic status (SES) and vaccination uptake, grouped by structural and organizational factors of primary care. Differences between social groups in this table were denoted as + if the study reported a difference of at least 5% in uptake between high and low SES and this difference was found to be statistically significant.

**Table 2:** Number of results on vaccination coverage in relation to parental SES and primary care models

	Total # of studies	Parental education			Parental occupation			Parental income			Area level SES		
		+	–	0	+	–	0	+	–	0	+	–	0
Type of Primary care													
Hierarchical with WBC	3	1								2			2
Hierarchical without WBC	5	1	1	2			2	1			1		
Non-Hierarchical with WBC	1			1						1			
Non-Hierarchical without WBC	6	2		3			1						

If a study had more than one SES measure, it was then recorded twice in the above table. For ex. if the vaccination coverage was analyzed both with respect to parental education and household income, the study was recorded both under the 1st and 3rd columns

+: positive association between SES level and vaccination coverage: higher SES, higher vaccination coverage, −: negative association between SES level and vaccination coverage: higher SES, lower vaccination coverage, 0: No association between SES and vaccination, socially equitable outcome

## Results

**Fig. 1:**
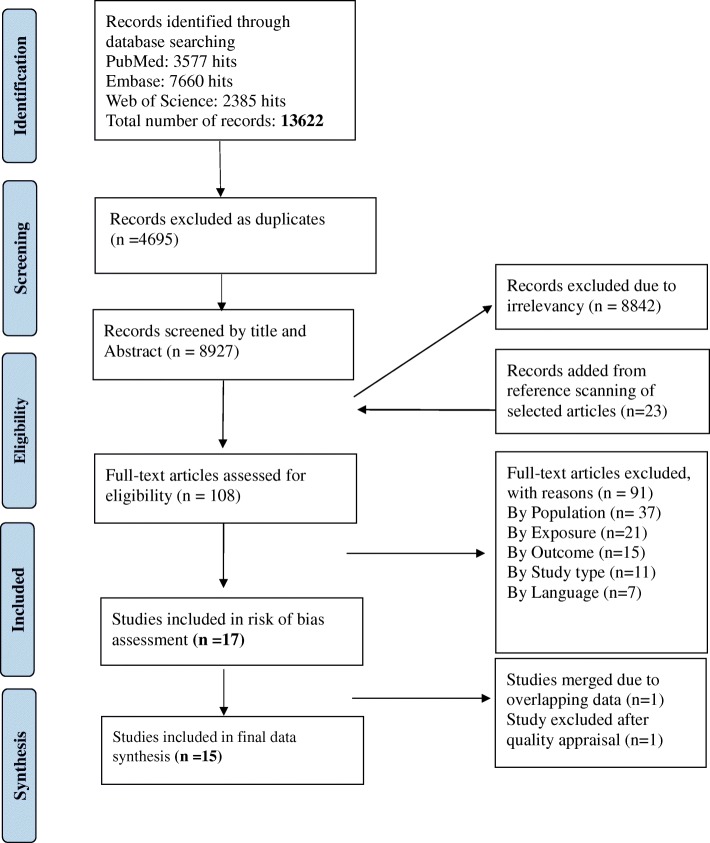
Flowchart of the systematic search process (Following Prisma Guidelines)

A total of 8927 unique articles were screened by title and abstract, 108 articles were found as eligible for reading in full-text after which 17 studies were selected for data extraction. Two articles from Belgium [12, 13] had overlapping data sets and were therefore merged into one study, in the process of data synthesis. The risk of bias was assessed to ensure the quality of included studies (Additional file 2). High risk of a bias, in a Greek study of preschool children in nurseries, led to the exclusion of one study [14] leaving 15 studies in the final synthesis of the results. A summary of this process is shown in Fig. 1.

### Description of included studies

The review included studies from ten European Countries (Belgium, France, Germany, Greece, Ireland, Italy, Netherlands, Spain, Sweden, and United Kingdom) and Australia. Germany, Ireland, UK and Greece contributed with more than one publication, whereas the rest of the countries contributed with a single study to the review. 10 studies had regional samples of the population while the rest had national samples. The details of the 15 studies in the analysis are presented in Table 1.

Population size varied from 630 [15] to 180,456 [16] individuals between the studies. The date of publication spanned the period of 2007–2017. The total time range for data collection was 1995 to 2012, with all studies collecting most of their data after 2000. Older studies were primarily based on surveys of comparatively small regional samples of populations, while many of the more recent studies were based on entire register information of national cohorts or random samples of such cohorts.

The indicators of SES varied considerably between studies. The majority of the studies examined the association between parental education and vaccination uptake. Several studies had results stratified by parental income and only a few by parental occupation or area level SES (Table 2).

### The social patterns of vaccine uptake

Across the eleven countries included in the study only four countries (Sweden, Netherlands, Spain and Italy) reached herd immunity level of 93–95% for MMR and/or 85% for DTP. A nationally representative study from Greece showed the level of vaccination uptake of MMR and DTP combined to be as low as 63% and in the case of France, the overall vaccination coverage was found to be even lower, 40%.

As seen in Table 2, out of twenty-one results, fourteen showed no association between socioeconomic level and vaccination coverage. In all the studies that showed some form of social inequity, the significant differences did not follow a gradient but were rather specific to a single social group, except for a study from UK by Hungerford et al. [17].

#### Hierarchical model with well-baby clinics

Among the three studies in countries with a hierarchical model and well-baby clinics, the studies by Wallby et al. from Sweden [18] and Pearce et al. from Australia [19] showed equitable outcomes for vaccination coverage regarding parental household income. The results from a Dutch study by van Lier et al. [16] showed no differences in MMR and DTP coverage between areas with high and low SES. In the case of the Australian study, parental education was found to be an important factor for the lowest educated group: 17.4% of the children with mothers less than ten years of education had incomplete immunization as compared to 6.7% of children of mothers with a degree.

#### Hierarchical model without well-baby clinics

The studies from countries with hierarchical model of health care without well-baby clinics showed indication of unequitable vaccination coverage for parental income and area level SES. An interesting finding was the negative association for parental education in a nationally representative study by Pearce et al. from UK [20] that showed the children of mothers with higher education to be 1.41 times more likely to be unimmunized compared to uneducated mothers. An Irish study by Doherty et al. [21], that analyzed DTP coverage in a nationally representative sample found equitable uptake in regards to maternal education. In the same study, vaccination coverage was found to be equitable across parental occupational groups, which was also the finding of a small regional study by Borras et al. from Spain [15].

#### Non-hierarchical model with well-baby clinics

There was only one study from a country with a non-hierarchical primary care model that included well-baby clinics for the youngest children. The results from this regional Belgian study by Theeten et al. [12] and Vandermeulen [13], with a relatively small sample size, suggested equitable vaccination coverage with respect to parental education and income.

#### Non-hierarchical model without well-baby clinics

Two of the six studies from countries with non-hierarchical model that lack well-baby clinics showed inequities in vaccination coverage, with largest gaps between social groups. A nationally representative study by Fonteneau et al. from France [22] showed uptake of MMR in children of farmers to be 33% and 42–46% in other occupational groups. Similarly, a nationally representative study by Danis et al. from Greece [23] demonstrated that vaccination uptake was as low as 54% in children of low educated mothers compared with 69% in children of mothers with a university degree. Two studies from Germany by Mikolajczyk et al. [24] and Poethko-Muller et al. [25] as well as an Italian study by Anello et al. [26], however, found equitable vaccination coverage based on parental education levels.

## Discussion

In this systematic review, we have investigated the social patterns of vaccine uptake in Europe and Australia in relation to their organisation of primary care services. The diversity of social indicators used in the studies we identified did not allow us to perform a meta-analysis, but nonetheless some tentative patterns emerged. All studies in primary care models with well-baby clinics showed high overall vaccination coverage and six out of the seven studies demonstrated equitable uptake of vaccination. Lowest levels of overall vaccination coverage and relatively wider gaps based on parental education and occupation were observed in Greece and France, countries with non-hierarchical primary care organization that did not have well-baby clinics. Studies from Germany and Italy, however, countries with a non-hierarchical model of primary care, showed a high coverage and quite equitable uptake of vaccine, indicating that it is possible to obtain satisfactory vaccine uptake also with a non-hierarchical model of primary care.

Our analysis suggests that providing preventive health services in a special organisation within primary care may increase equity in vaccine uptake for preschool children. Although we acknowledge that there can be multiple factors in play, we believe well-baby clinics deserve special attention due to its potential in meeting the quality criteria for primary care services suggested by Starfield [27]. The four quality criteria: access, longitudinality, comprehensiveness and coordination can be used as a framework to understand the mechanisms behind the role of the well-baby clinics. It seems likely that this special organization facilitates easy access when vaccination does not have to compete with other priorities and provides a continuity in care that leads to a trustful relation between health care provider and family by repeated visits to the same nurse from the neonatal period until school entry. Trust to the healthcare providers has previously been found to have positive association with vaccination uptake [7, 23]. Compared to more fragmented models of preventive health where different health organizations deliver different services, the well-baby clinics provide a comprehensive platform for various preventive interventions and coordinate referrals to other health care providers motivated by screening procedures or negative vaccine reactions.

Previous research on interventions to reduce social inequalities in vaccination uptake have primarily focused on their implementation within one specific primary health care model, for example the NHS model in the UK. A recent systematic review found complex, and locally designed, interventions to be more effective in buffering disadvantages [7]. To some extent, these interventions resulted in a model of preventive care that is similar to the well-baby clinics since they enabled easy access and continuity in relation between healthcare provider and family. This suggests that taking a broader perspective might be beneficial in future developments in this field, where changes in the overarching primary care model, may be a more effective way to improve equity in vaccination uptake than to implement small-scale interventions within the existing primary care models.

The potential of well-baby clinics as a platform for targeted interventions has been shown in a recent Swedish intervention study [28, 29]. In this study, guided by a “proportionate universalism” approach [30], all families with infants in a disadvantaged neighbourhood were offered an intensified home visiting program by the well-baby clinic in collaboration with the social services in the area. The preliminary outcomes were promising with diminishing inequities in levels of vaccination coverage as one result [28].

Primary health care organizations that are based on the non-hierarchical model, such as those of Belgium, France, Greece and Germany, all provide a considerable freedom for primary care physicians to choose their area of practice; and there is comparatively limited influence of the national or regional state on its regulation and financing. Our systematic review shows that this type of health care organization in some countries is associated with considerably lower levels of overall vaccination coverage and higher social inequities with respect to parental occupation and education, but not in all countries with this model. France is an example of such a model with a low uptake. The French model is dominated by privately owned clinics and requires parents to buy the vaccination themselves with co-payments, which are later compensated by the government. These are possibly the two main driving forces for the low vaccination levels [31]. Italy is an example of a country with a similar primary model that has succeeded in obtaining a high uptake of vaccine by having an ambitious national vaccine policy [32].

Health care reform is currently underway in many European countries with a National Health Service, such as the UK, Spain and Sweden. One major shift due to the reforms is facilitation of the establishment of new private outpatient practices reimbursed by public funds at locations chosen by the health care professionals themselves. The changes also include increases in the proportion of private providers, application of market-based mechanisms, the promotion of a patient choice agenda and changes to resource allocation systems. Thus, the reforms are moving these primary care models closer to the non-hierarchical model. In countries with this model, studies in the adult population have shown that such changes lead to increased inequity in utilisation of primary care [33]. Consequences of these changes for equity in children’s access to preventive care should routinely be monitored.

### Strengths and limitations

#### Strengths

This systematic review is pioneering a systems approach in assessing social inequities in vaccination coverage for infants and pre-school children across European countries and Australia. As opposed to earlier systematic reviews where social differences in childhood vaccinations have been analyzed within a specific health care system [3, 7], this review takes a broader perspective in analyzing the social differences in vaccination coverage by comparing structural and organizational aspects of primary care and preventive services across countries. The specific focus on infants and pre-school age children made it possible for us to focus on the preventive health services for the youngest children, a most important age group for health promotion in a life course perspective.

The authors of this paper followed the universally accepted and accredited PRISMA statement and PICOS checklist in selection and synthesis of the results. Furthermore, an assessment on risk of bias was carried out to make sure a certain level of quality in the studies that were used to draw conclusions.

#### Limitations

The main limitation of this review is the heterogeneity of the socio-economic indicators across studies and national contexts. Not only are diverse indicators used, different categorisations of the same indicator are also common. Furthermore, the validity of a social indicator is bound to a certain societal context, making cross-country comparisons of social gradients difficult to interpret. Thus, differences between countries in the magnitude of social gradients should be interpreted with caution.

Another limitation is the comparatively long time period included in the review, made necessary by the scarcity of the studies available. Thus, health care reform during more recent years may have affected our analysis so that some of our conclusions are no longer relevant.

A heterogeneity in study designs should also be kept in mind in the interpretation of our results. Surveys and longitudinal cohort studies typically have a selective attrition of socially disadvantaged segments of the population, while this is less of a problem with register data [34].

This study focuses only on two structural and organizational aspects of PCSs, namely the degree of freedom of the primary care provider and the existence of well-baby clinics. It is possible that other aspects of the organisation of pediatric primary care are of importance, such as the type of physician involved in the preventive health care (i.e. a pediatrician or a general practitioner). However, in our study this aspect does not explain the observed inequity patterns of vaccination coverage. Further studies are needed to evaluate other structural and organizational aspects of vaccination delivery such as integration of services, national commitment in vaccination policies, and use of registers. Nevertheless, we believe that our results raise important issues for further consideration and research.

Finally, we have to acknowledge the limitations of vaccine uptake as an indicator for access to preventive health services that provides vaccinations. Some parents make a conscientious choice not to vaccinate their children. This has been a particularly pertinent problem with measles vaccine in the aftermath of the Wakefield article in the Lancet about a possible connection with this vaccine and autism [35], an article that has later been convicted of fraud. This article was published in 1998 and may thus have had an influence on the results of the relatively older studies in the review, such as the British study by Pearce et al. [20] that showed reversed educational gradient in vaccination uptake.

## Conclusion

When analyzed through a lens of structural and organizational factors, our review found some evidence for the role of well-baby clinics in providing more equitable vaccination services for children. Countries with well-baby clinics showed higher overall rates of vaccination, and there were less social inequities. Lowest vaccination coverage rates and high gaps due to parental occupation and education were observed mostly in systems with non-hierarchical systems that also lacked well-baby clinics.

The analysis of the results was greatly hampered by low number of studies that examine social inequities in vaccination uptake in Europe. This knowledge gap shows us where more research and monitoring is needed to inform primary care models for children with regards to equity.

## Additional files

Additional file 1: The search string used in Pubmed. (DOCX 13 kb)

Additional file 2: Assessment of Risk of Bias. (DOCX 13 kb)

## Abbreviations

DTP: Diphteria-Tetanus-Pertusis
EEA: European Economic Area
EFTA: European Free Trade Association
MMR: Measles-Mumps-Rubella
MOCHA: Models of Child Healthcare Appraised
PCSs: Primary care services
SES: Socio-economic status
WBC: Well-baby clinics
