# Supplementary material for: Social inequities in vaccination coverage among infants and pre-school children in Europe and Australia – a systematic review
Source: BMC Public Health. 2019 Mar 12;19:290. doi: 10.1186/s12889-019-6597-4 (PMC6417277; doi:10.1186/s12889-019-6597-4)
Supplement: Supplementary file 1 — The search string used in Pubmed. (DOCX 13 kb) [file 12889_2019_6597_MOESM1_ESM.docx]

## **Additional file 1**

The search string used in Pubmed:

- Vaccination

(Vaccination [MeSH Terms]) OR (“vaccination” [Title/Abstract]) OR (“vaccine” [Title/Abstract]) OR( “vaccines” [Title/Abstract]) OR (immunisation [Title/Abstract]) OR (immunization [Title/Abstract]) OR (“vaccinated” [Title/Abstract]) OR (“immunized” [Title/Abstract]) OR (“immunised” [Title/Abstract])

**AND**

- Children and infants

(child[MeSH Terms]) OR (minors[MeSH Terms]) OR (child health services[MeSH Terms]) OR (infant[MeSH Terms]) OR (child[Title/Abstract]) OR (children[Title/Abstract]) OR (infant[Title/Abstract]) OR (infants[Title/Abstract])

**AND**

- Social adversity

(socioeconomic factors[MeSH Terms]) OR (transients and migrants[MeSH Terms]) OR ("social class"[Title/Abstract]) OR ("social status"[Title/Abstract]) OR ("socioeconomic factors"[Title/Abstract]) OR (ethnic*[Title/Abstract]) OR (socioeconomic[Title/Abstract]) OR ("socio economic"[Title/Abstract]) OR ("socio-economic"[Title/Abstract]) OR ("socio-demographic "[Title/Abstract]) OR ("sociodemographic"[Title/Abstract]) OR ("socio demographic"[Title/Abstract]) OR (social disadvantage*[Title/Abstract]) OR ("household income"[Title/Abstract]) OR (immigrant*[Title/Abstract]) OR (migrant*[Title/Abstract]) OR (refugee*[Title/Abstract]) OR (asylum seek*[Title/Abstract]) OR (social disparit*[Title/Abstract]) OR (inequity [Title/Abstract]) OR (inequities [Title/Abstract]) OR ("Socially disadvantaged"[Title/Abstract]) OR ("Social disadvantage"[Title/Abstract]) OR ("social inequalities" [Title/Abstract])OR ("social inequality" [Title/Abstract])
