# Supplementary material for: Social inequities in vaccination coverage among infants and pre-school children in Europe and Australia – a systematic review
Source: BMC Public Health. 2019 Mar 12;19:290. doi: 10.1186/s12889-019-6597-4 (PMC6417277; doi:10.1186/s12889-019-6597-4)
Supplement: Supplementary file 2 — Assessment of Risk of Bias. (DOCX 13 kb) [file 12889_2019_6597_MOESM2_ESM.docx]

## **Additional file 2**

Assessment of Risk of Bias

1. Was selection bias minimized in the selection of study participants from the sampled population?
2. Is the information on socioeconomic variables obtained from sources that would minimize attrition bias (i.e. from registers or validated questionnaires)?
3. Is the information on vaccination status obtained from sources that would minimize information bias (i.e. from registers/health records/vaccination cards or validated questionnaires)?

Studies that used regional/national registers for obtaining information on socioeconomic variables and vaccination status were ranked as having low risk of bias. Studies that used survey data were ranked as medium risk of bias. Studies that had major selection bias due to selective inclusion of study participants were ranked as high risk of bias, and were excluded from the synthesis of results.
